# Zonular fibre Insertion-to-Limbus Distance (ZLD): normative data to assess lens position and diagnose ectopia lentis

**DOI:** 10.1007/s10792-024-03163-0

**Published:** 2024-06-24

**Authors:** Max Rohrberg, Vanessa Lussac, Daniel J. Salchow

**Affiliations:** Department of Ophthalmology, Charité – University Medicine Berlin, Campus Virchow Clinic, Augustenburger Platz 1, 13353 Berlin, Germany

**Keywords:** Ectopia lentis, Lens subluxation, Marfan syndrome, Zonular fibres, Limbus, Anterior chamber, Anatomy, Diagnostic parameters

## Abstract

**Purpose:**

Subluxation of the crystalline lens (Ectopia Lentis, EL) can lead to significant visual impairment and serves as a diagnostic criterion for genetic disorders such as the Marfan syndrome. There is no established criterion to diagnose and quantify EL. We prospectively investigated the distance between the zonular fibre insertion and the limbus (ZLD) in healthy subjects as a parameter to assess the position of the lens, quantify EL and provide normative data.

**Methods:**

This prospective, observational, cross-sectional study includes one-hundred-fifty eyes of 150 healthy participants (mean age 28 years, range 4–68). Pupils were dilated with tropicamide 0.5% and phenylephrine 2.5% eyedrops. ZLD was measured in mydriasis at the slit lamp as the distance between the most central visible insertions of the zonular fibres on the lens surface and the corneoscleral limbus. Vertical pupil diameter (PD) and refractive error were recorded. If zonular fibre insertions were not visible, the distance between limbus and the pupillary margin was recorded as ZLD.

**Results:**

145 right and 5 left eyes were examined. 93% of study subjects were Caucasian, 7% were Asian. In eyes with visible zonular fibre insertions (n = 76 eyes), ZLD was 1.30 ± 0.28 mm (mean ± SD, range 0.7–2.1) and PD was 8.79 ± 0.57 mm (7.5–9.8). In the remaining 74 eyes, ZLD was 1.38 ± 0.28 mm (0.7–2.1), and PD was 8.13 ± 0.58 mm (6.7–9.4). For all eyes, ZLD was 1.34 ± 0.29 mm (0.7–2.1), and PD was 8.47 ± 0.66 mm (6.7–9.8). Refractive error and sex did not significantly affect ZLD. Smaller PD and older age were associated with larger ZLD (*P* < 0.001 and *P* = 0.036, respectively).

**Conclusion:**

Average ZLD was 1.34 mm in eyes of healthy subjects. Older age correlated with larger ZLD. These normative data will aid in diagnosing and quantifying EL.

## Introduction

The crystalline lens focuses light to provide a sharp image on the retina. Ectopia lentis (EL) describes a displaced lens, including milder (subluxation) and complete (luxation) forms. EL can lead to significant visual impairment. It may be caused by trauma, inflammation, pseudoexfoliation syndrome and connective tissue disorders including Marfan syndrome (MFS) [1, 2]. Complications of EL include decreased vision, glaucoma, uveitis and retinal detachment [3–7].

Systems to grade EL have been described. Waiswol et al. [8] graded EL at the slit lamp in relation to the undilated pupil. Examination without pupil dilation precludes mild EL from being detected. The “grading in ectopia lentis” classification was described by Chandra and colleagues [9]. This method requires pupil dilation, and it specifies direction and extent of EL. Chandra et al. [9] assessed the “displacement” in the z-axis (anterior–posterior direction) and “subluxation” frontal plane. The extent is graded as 1 (edge of the lens has not passed the pupillary axis) und 2 (edge of the lens has passed the pupillary axis). This system is based on photographs and seems reproducible, but we are not aware of any studies evaluating it clinically. Zech and coworkers [10] used a Goldmann contact lens on the cornea to grade EL in a cohort of MFS patients. This method provides information on the antero-posterior lens position and grades EL (0 = normal position, 5 = luxation of the lens). Complete luxation of the lens is rare [1] and most cases of EL fall into grades 2 to 4 according to this system. While each grading system has advantages, neither allows quantification of EL on a continuous scale and normative values are lacking. A classification that can be performed at the slitlamp would greatly facilitate diagnosis and quantification of EL.

We describe a novel parameter using the corneoscleral limbus as reference, from which the distance.

to the zonular fibre insertion on the lens is measured in mydriasis at the slit lamp (zonular fibre.

insertion-to-limbus distance, ZLD), and provide normative values.

## Methods

### Subjects

This prospective study included patients presenting to the Eye Clinic, at Charité University Medicine Berlin—Virchow Hospital Campus, between July 2021 to February 2023, as well as healthy volunteers. Subjects enrolled consented in writing and had no intraocular pathologies and no intraocular comorbidities other than refractive error.

Patients with diagnoses associated with EL (e.g. certain connective tissue disorders, previous ocular trauma or intraocular surgery) were excluded. The study was in accordance with the Charité statutes for ensuring good scientific practice. An Institutional Review Board (IRB)/Ethics Committee approval was obtained. The research adhered to the tenets of the Declaration of Helsinki.

### Measurements

**Fig. 1:**
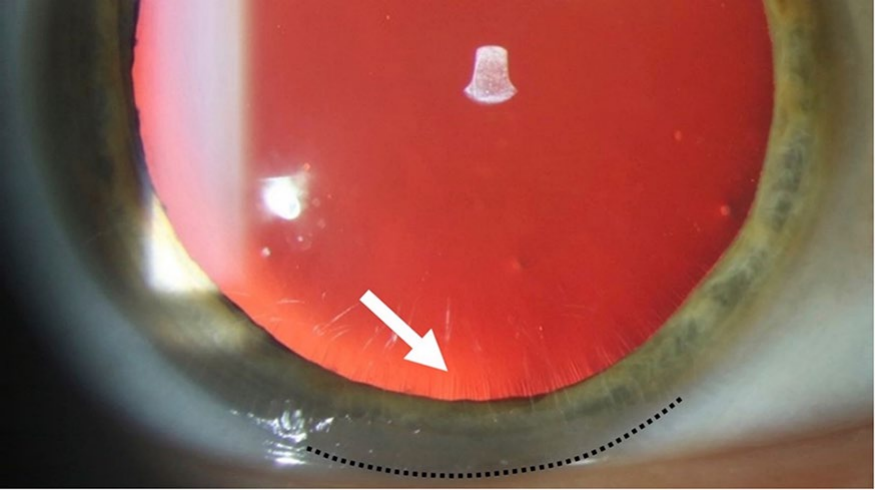
Slit lamp photograph of a healthy subject with visible zonular fibre insertions (white arrow) from 4 to 8 o’clock, vertical pupil diameter = 9.1 mm. To minimize variability when measuring manually at the slit lamp, the halfway point between the clear cornea and the white sclera should be used (black dotted line)

Pupils were dilated with tropicamide 0.5% and phenylnephrine 2.5% eye drops given twice 5 min apart; 4 participants received one set of drops, all had visible zonular fibre insertions. Refractive error was measured with autorefraction (RM 8800 Topcon, Itabashi, Japan and ARK-1 s Nidek, Aichi, Japan) before the pupils were maximally dilated. When mydriasis was maximal, PD measured using a handheld photo-refractometer (Plusoptix A09, Plusoptix, Nuremberg, Germany), which also measures pupil diameter. Zonular fibre insertions are best seen in retroillumination (Fig. 1). ZLD was measured using the slit lamp (BX 900, Haag-Streit, Köniz, Switzerland, and SL 120, Carl Zeiss Meditech, Jena, Germany) after calibration of the slit height in the focal plane. ZLD was measured once per subject and was recorded as the distance between the limbus and the zonular fibre insertion closest to the centre of the lens. The location of this measurement was indicated using clock hours (e.g. ZLD 1.3 mm @ 6 o’clock). If zonular fibre insertions were broadly visible, the centre of the area of visible zonular fibre insertions was indicated. Because the limbus is a transition zone rather than a sharply demarcated line, the middle of the grey band between clear cornea and white sclera was used as the limbus.

**Fig. 2:**
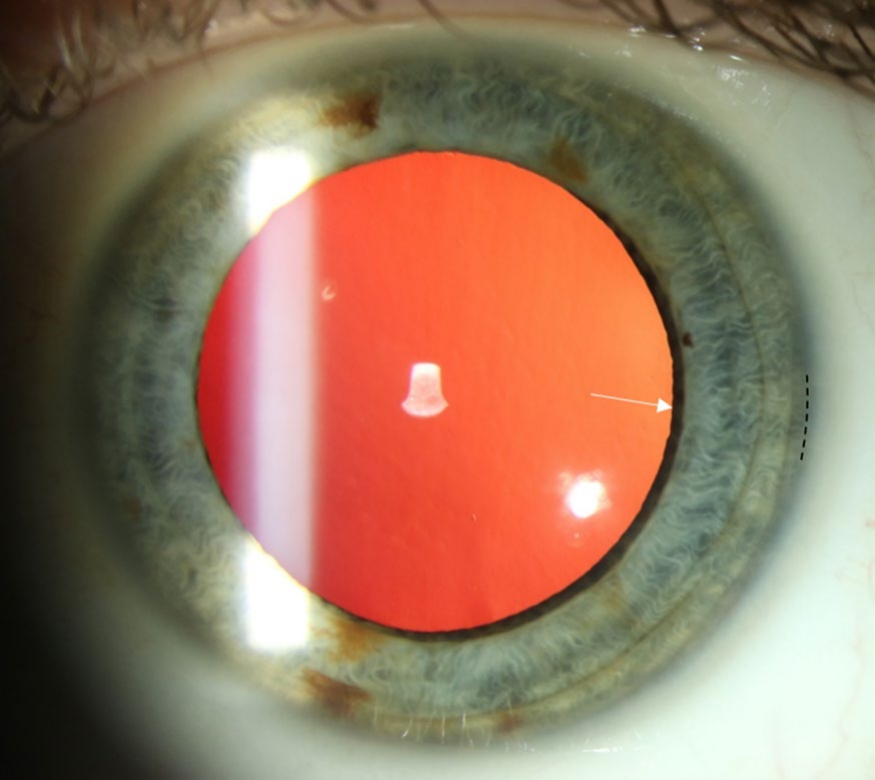
Slit lamp photograph of a healthy subject without visible zonular fibre insertions (white arrow), vertical pupil diameter = 7.8 mm. To minimize variability when measuring manually at the slit lamp, the halfway point between the clear cornea and the white sclera should be used (black dotted line)

In eyes without visible zonular fibre insertions in mydriasis, the largest distance between the limbus and the pupil margin, representing the minimal ZLD, was recorded. For purposes of this study eyes were considered myopic if the refractive error was at least -1 dioptre (Fig. 2).

## Statistics

Mean values, standard deviation and range were calculated, comparisons of variables between groups were performed using the unpaired Student’s t test. The chi-square test was used to compare categorical variables. To analyse the effect of PD, refractive error, age, and sex on ZLD, univariate regression analysis was done. Statistical operations were performed using SPSS (version 27.0, IBM Corp., Armonk, NY).

## Results

One hundred fifty eyes (145 right, 5 left eyes) of 150 subjects were examined. In approximately half of them, zonular fibre insertions were visible in mydriasis (Table 1). Mean ZLD (± standard deviation) was 1.34 ± 0.29 mm (95% CI, 1.29–1.38 mm). Zonular fibre insertions were more often visible in myopic than in non-myopic eyes (64% versus 44%, *P* = 0.021), and PD of myopic eyes was greater than that of non-myopic eyes (Table 2).

**Table 1:** Demographics of study subjects and comparison between eyes with visible zonular fibre insertions in mydriasis versus those without

Parameter	All	Zonular Fibre Insertions Visible	Zonular Fibre Insertions Not Visible
n	150	76	74
*Age (yrs)*			
Mean	28	27	28
Range	4–68	4–63	11–68
*Sex*			
Female	92 (61%)	48	44
Male	58 (39%)	28	30
*Race/Ethnicity*			
Asian	11 (7%)	6	5
Caucasian	139 (93%)	70	69
Right eyes	145	72	73
Left eyes	5	4	1
*PD*			
Mean ± SD	8.47 ± 0.66	8.79* ± 0.57	8.13* ± 0.58
Range	6.7–9.8	7.5–9.8	6.7–9.4
95% CI	8.36–8.57	8.66–8.92	7.99–8.26
*ZLD*			
Mean ± SD	1.34 ± 0.29	1.30* ± 0.28	1.38* ± 0.28
Range	0.7–2.1	0.7–2.1	0.7–2.1
95% CI	1.29–1.38	1.23–1.36	1.32–1.45

PD = pupil diameter in mm; SD = standard deviation of the mean; ZLD = zonular fiber insertion-to-limbus distance in mm; 95% CI = Confidence Interval;

*P < 0.001 for PD and *P* = 0.035 for ZLD

**Table 2:** Zonular fibre insertion-to-limbus distance and pupil diameter after pharmacologic dilation in myopic and non-myopic eyes

Parameter	Myopic Eyes	Non-Myopic Eyes	*P*-Value
n	50	100	
*Age (yrs)*			
Mean	25	29	
Range	11–51	4–68	
*Refractive error*			
Mean ± SD	− 3.15 ± 1.85	0.28 ± 0.84	
Range	− 8 to − 1	− 0.75 to + 5.25	
*Zonular fibre insertions*			
Not visible	18* (36%)	56* (56%)	
Visible	32* (64%)	44* (44%)	°0.021
PD	8.62* ± 0.64	8.39* ± 0.66	*0.019
ZLD	1.34 ± 0.29	1.34 ± 0.29	0.468

Refractive error in dioptres; SD = standard deviation of the mean;

*P < 0.05; °*P-*value for chi-square test; *P-*values of unpaired t-test

The location of visible zonular fibre insertions was documented in 71 / 76 eyes with visible zonular fibre insertions. In 66.7%, they were visible in the inferior quadrant, in 17.0% nasally, in 10.9% temporally, and in 5.4% superiorly. Most eyes had zonular fibre insertions which were wider than one clock hour. Larger PD correlated with lower ZLD (Table 3, *P* < 0.001). Refractive error and sex did not affect ZLD, older age correlated with greater ZLD (*P* = 0.036). There was nearly a significant association between older age and smaller PD (*P* = 0.068).

**Table 3:** Univariate regression analysis of subject characteristics regarding their effect on zonular fibre insertion-to-limbus distance

Parameter	PD	Refractive Error	Age	Sex
All (n = 150)	< 0.001*	0.997	0.036*	0.464
Zonular fibres not visible	0.001*	0.519	0.301	0.528
Zonular fibres visible	< 0.001*	0.676	0.073	0.578

*P-*values of univariate regression analysis

*P < 0.05

## Discussion

While severe EL is easily identified, milder forms are harder to find. Quantification of EL is limited by the lack of a scaling system that uses a continuous numeric parameter and provides normative data [8–10]. Measuring ZLD is a non-invasive, non-contact method, facilitating its use in patients with limited cooperation, e.g. in children. Pharmacological mydriasis is necessary to identify zonular fibre insertions unless EL is severe. The corneoscleral limbus and the insertion of the zonular fibres on the lens surface serve as landmarks to measure ZLD. The limbus as the transitional zone from the clear cornea to the white sclera [11] has an average width of 1 mm in (range 0.7–1.4 mm) in human cadaver eyes [12]. Variability when measuring the ZLD can be minimized by choosing the centre of the limbus. Larger variability of identifying the limbus has been reported when the white-to-white distance is measured automatically with computer-based instruments. There is limited agreement between the measurements with different instruments, but good repeatability for each method when measuring the white-to-white distance automatically. [13] Because ZLD is measured at the plane of limbus, the measuring slit can simultaneously be focused on the anterior lens surface. Care should be taken to calibrate the measuring slit at the focal plane of the slit lamp to ensure accurate measurements.

If zonular fibres are ruptured, their insertions may not be visible on the lens surface. This can be accounted for by using the lens edge to measure ZLD and adding a “+” after the value, indicating more severe EL (e.g. ZLD 3.0 mm @ 6 o’clock +).

Detecting EL is important to avoid visual impairment and to identify a potentially serious underlying disorder. EL is a major diagnostic criterion for MFS [14], caused by mutations in the fibrillin-1 gene [15, 16], with life- threatening cardiovascular manifestations. Other entities associated with EL include homocystinuria, which may be complicated by thromboembolic events, osteoporosis and developmental delay, as well as ocular conditions such as uveitis, pseudoexfoliation syndrome, trauma and previous surgery.

Our results suggest that ZLD is not affected by sex. Older age was associated with larger ZLD, most likely because older age was associated with smaller PD. Refractive error did not affect ZLD significantly, but in myopic eyes zonular fibre insertions were more often visible than in non-myopic eyes, possibly because their pupils dilated more.

Larger PD was associated with lower ZLD. This can be expected, because if the pupil is small, more peripheral zonular fibre insertions are covered by the iris. On the other hand, a larger pupil allows identification of these more peripheral insertions, resulting in a lower ZLD. In eyes without visible zonular fibre insertions, the distance of the pupil margin was used to measure the distance to the limbus. In these eyes, ZLD may be smaller than the recorded surrogate. Considering that the actual ZLD is equal to or less than the value recorded in these eyes, the average ZLD reported in this study may be overestimating ZLD in healthy subjects. However, the average difference of ZLD between eyes with visible zonular fibre insertions and those without was small (< 1 mm).

We included one eye per subject in our analysis, and 97% of eyes studied were right eyes. Whether ZLD is similar in both eyes of a healthy subject cannot be answered based on our study results. Most of our subjects were Caucasian and our results may not apply to all populations.

Another proposed system to grade EL at the slitlamp [8] uses the undilated pupil, and therefore cannot detect mild degrees of EL. Another system reported by Chandra and colleagues [9] requires mydriasis and specifies direction and extent of EL using the two parameters “displacement” in the z-axis (anterior–posterior direction) and “subluxation” in the frontal plane. This system is based on photographs, limiting its use in clinical practice. Normative values are not available for either method. Using a Goldmann contact lens on the cornea to grade EL [10] may be a sensitive method in MFS patients. Its use in children and uncooperative patients however is limited.

These previously proposed systems quantify EL on categorical scales. ZLD allows measuring EL in mm on a continuous scale, facilitating longitudinal follow-up and statistical analysis. ZLD can be measured at the slit lamp without the need for other instruments or photographs. Photographic assessment of ZLD is optional and shows good correlation with manual measurements in preliminary studies (data not shown). It is conceivable that severe EL, with posterior subluxation of the lens, may introduce variability into the measurement of ZLD. This can be addressed in future studies. In summary, ZLD is a novel parameter for quantifying EL, the normative data provided facilitate its diagnosis. It offers a continuous numeric scale to quantify EL. Future research may focus on quantifying EL in patients with subluxated lenses and follow them longitudinally.
